# Association between *eNOS* rs1799983 polymorphism and hypertension: a meta-analysis involving 14,185 cases and 13,407 controls

**DOI:** 10.1186/s12872-021-02192-2

**Published:** 2021-08-09

**Authors:** Jikang Shi, Siyu Liu, Yanbo Guo, Sainan Liu, Jiayi Xu, Lingfeng Pan, Yueyang Hu, Yawen Liu, Yi Cheng

**Affiliations:** 1 Department of Epidemiology and Biostatistics, School of Public Health, Jilin University, Changchun, 130021 China; 2 Department of Children and Adolescence Health, School of Public Health, Jilin University, Changchun, 130021 Jilin China; 3 The Cardiovascular Center, The First Hospital of Jilin University, Changchun, 130021 China

**Keywords:** Hypertension, *eNOS*, rs1799983, Polymorphism, Meta-analysis

## Abstract

**Background:**

Essential hypertension is a complex disease determined by the interaction of genetic and environmental factors, *eNOS* is considered to be one of the susceptible genes for hypertension. Our study aimed to evaluate the association between *eNOS* rs1799983 polymorphism and hypertension, and to provide evidence for the etiology of hypertension.

**Methods:**

Case–control studies of *eNOS* rs1799983 polymorphism and hypertension were included by searching PubMed, Embase, Web of Science, Medline, Scopus, WanFang datebase, Vip datebase, and CNKI database according to PRISMA guideline. Eligible data were extracted and pooled, and were analyzed using R software based on five different genetic models.

**Results:**

A total of 60 eligible articles involving 14,185 cases and 13,407 controls were finally selected. We found significant association between *eNOS* rs1799983 polymorphism and hypertension under any genetic model (T vs G: *OR* = 1.44, 95% CI 1.26–1.63; GT vs GG: OR 1.34, 95% CI 1.18–1.52; TT vs GG: OR 1.80, 95% CI 1.41–2.31; GT + TT vs GG: OR 1.42, 95% CI 1.25–1.63; TT vs GG + GT: OR 1.68, 95% CI 1.35–2.08; GT vs GG + TT: OR 1.24, 95% CI 1.11–1.40).

**Conclusions:**

We found that *eNOS* rs1799983 polymorphism is associated with the increased risk of hypertension under any genetic model. Moreover, investigations of gene–gene and gene-environment interactions are needed to give more insight into the association between *eNOS* rs1799983 polymorphism and hypertension.

**Supplementary Information:**

The online version contains supplementary material available at 10.1186/s12872-021-02192-2.

## Background

Essential hypertension (EH) is a complex disease determined by the interaction of genetic and environmental factors, and EH is regarded as a predisposing risk factor for many diseases, such as myocardial infarction, stroke, and chronic renal failure [1]. So far, the pathogenesis underlying hypertension is still unclear in spite of the in-depth research being conducted on the mechanism of EH. However, increasing evidence supports the theory that genetic factors are a determinant of hypertension to a large extent [2], thus it is pivotal to identify susceptible genes for prevention, diagnosis, and treatment of hypertension [3]. Genes (*eNOS*) encoding endothelial nitric oxide synthase is considered to be one of the susceptible genes for hypertension because its enhanced production or enzyme bioavailability can lead to constitutive release of nitric oxide (NO) in endothelial cells, which is involved in blood pressure (BP) regulation [4].

Previous studies have shown that *eNOS* plays a critical role in regulating vascular tone and blood pressure. For example, overexpression of *eNOS* gene in transgenic mice leads to a significant decrease in blood pressure [5]. In addition, it was found that inhibition of *eNOS* gene in healthy individuals is associated with decreased levels of NO release and increased blood pressure [6].

The *eNOS* gene at 7q35-36 spans 21 kb, with 26 exons and 25 introns. There are about 10 polymorphic loci distributed in the promoter, exon, and intron of the *eNOS* gene. In these loci, the common mutation that leads to amino acid substitutions in mature proteins is G894T or Glu298Asp (rs1799983) mutations, in which base substitution of G to T will result in glutamic acid (Glu) being replaced at exon 7 by aspartic acid (Asp) at position 298 of the corresponding amino acid [7]. This genetic mutation reduces the production of NO and subsequently affects the development of EH [8].

A large number of articles have studied the association between *eNOS* rs1799983 polymorphism and hypertension; however, these results are still inconsistent. Recently, it is noted that new studies [9–12] on this theme have been published since the last meta-analysis published in 2017 [13]. Therefore, we included these newly published studies and conducted a further meta-analysis to investigate whether *eNOS* rs1799983 polymorphism is associated with hypertension.

## Materials and methods

### Literature search strategy

This meta-analysis was performed according to the statements in the Preferred Reporting Items for Systematic Reviews and Meta-Analyses (PRISMA) reporting standard [14]. Systematic literature search was performed in PubMed, Embase, Web of Science, Medline, Scopus, WanFang datebase, Vip datebase, and CNKI database up to October 30, 2020. Various combinations of terms used for searching were (“endothelial nitric oxide synthase” OR “nitric oxide synthase type III” OR “*eNOS*” OR “*NOS3*”) AND (“polymorphism” OR ‘‘variant” OR “mutation”) AND (“hypertension” OR “high blood pressure”). Moreover, we also retrieved and scrutinized related articles from the reference lists of literatures to replenish literatures that had not been identified in the initial search. A detailed form of the search strategy used in datebases was displayed in Additional file 1: Table S1.

### Inclusion/exclusion criteria

Studies included had to meet the following criteria: (1) case–control studies; (2) patients with essential hypertension were defined as cases, healthy subjects without hypertension were defined as controls; (3) evaluation of the association between *eNOS* rs1799983 polymorphism and hypertension. The exclusion criteria satisfied the followings: (1) case reports, review articles or cross-sectional studies; (2) duplicate articles; (3) secondary hypertension or gestational hypertension; (4) lack of sufficient information on genotype or allele frequencies.

### Data extraction and quality assessment

For each eligible study, the following data were extracted: name of first author, year of publication, region and ethnicity of study population, sample size, and numbers of *eNOS* genotype or allele in cases and controls. Hardy–Weinberg equilibrium (HWE) among the controls was calculated.

Quality of the included studies was evaluated using the Newcastle–Ottawa scale (NOS) [15] that has a “star” rating system consisting of selection, comparability, and exposure. The highest score of this rating system is 9 points. Moreover, the data extraction and quality assessment were performed by two investigators (Jikang Shi and Yanbo Guo) independently, and conflicts were resolved by discussing with the third investigator (Sainan Liu) if the results of two investigators didn’t reach an agreement.

### Statistical analysis

HWE was evaluated for control groups of each study using Goodness of fit Chi-square test, and *P* < 0.05 was considered as a significant deviation from HWE. The associations between *eNOS* rs1799983 polymorphisms and hypertension in this meta-analysis were measured based on five different genetic models including six comparisons: allelic model (T vs G), codominant model (GT vs GG and TT vs GG), dominant model (GT + TT vs GG), recessive model (TT vs GG + GT), overdominant model (GT vs GG + TT). Odds ratios (OR) and 95% confidence intervals (95% CI) were used to assess the strength of association between *eNOS* rs1799983 polymorphisms and hypertension. Q-statistic and *I*^2^-statistic were used to evaluate heterogeneity, random-effect models (DerSimonian and Laird methods) were used when heterogeneity existed (*I*^2^ ≥ 50% considered heterogeneity existed in between-study in this meta-analysis); otherwise, fixed-effect models (Mantel and Haenszel methods) were used. Subgroup analyses were performed by region, ethnicity, and HWE to detect main sources of heterogeneity and observe differences of the association in different groups. Sensitivity analysis was conducted to evaluate stability of our results by omitting each study at each time. Publication bias was estimated using funnel plots, and quantified by the Egger’s tests (*P* < 0.05 considered statistically significant publication bias) [16]. All data management and statistical analyses were performed using R Studio (Version 1.1.383) (RStudio, Inc., MA, USA) for Windows.

### Trial sequential analysis (TSA)

The risk of random error in traditional meta-analysis may increase because of the dispersed data and repeated significance testing [17, 18]. TSA was used to reduce the risk of type I error by adjusting threshold for statistical significance and to evaluate the required information size (RIS) and statistical reliability [19]. In our meta-analysis, trial sequential analysis software (TSA, version 0.9; Copenhagen Trial Unit, Copenhagen, Denmark, 2011) were performed, and additional studies were not needed when Z-curve crossed the trial sequential monitoring boundary or RIS has reached; otherwise, further studies were needed.

## Results

### Study characteristics

**Fig. 1:**
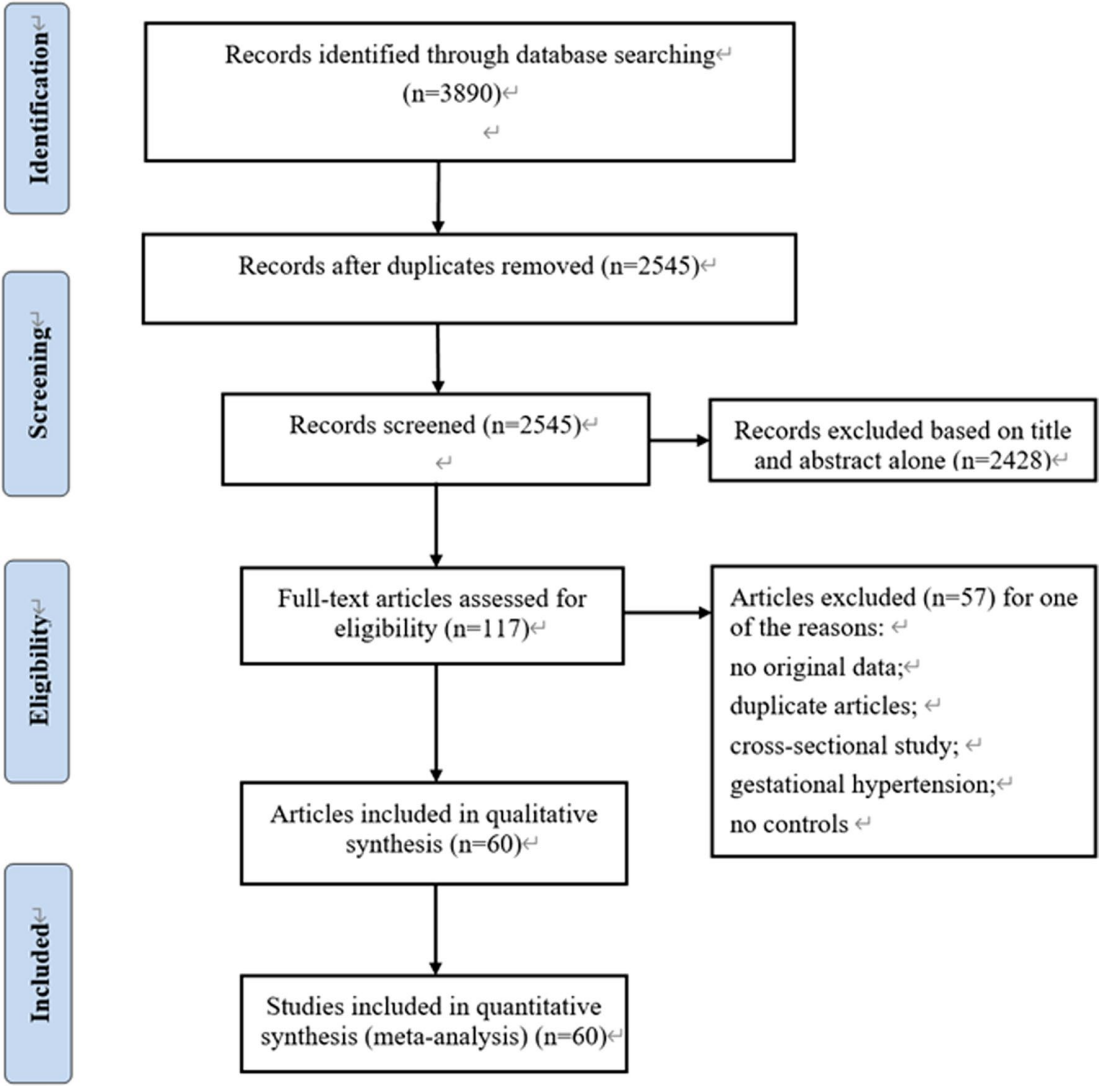
Flow chart of the process for literature identification and selection

A total of 60 eligible articles involving 14,185 cases and 13,407 controls were finally selected after strict screening on the basis of the inclusion and exclusion criteria, the protocol of literature search and selection is shown in Fig. 1, and the main characteristics and genotype distribution of the eligible studies are listed in Table 1.

**Table 1:** Main characteristics of the included studies

Study	Year	Region	Ethnicity	Sample size		Quality score	HWE Y/N	GG (n)		GT (n)		TT (n)	
				(Case/control)				Case	Control	Case	Control	Case	Control
Lacolley	1997	France	Caucasian	309/	123	7	0.250	140	35	122	67	47	21
Miyamoto	1998	Japan	Asian	218/	240	8	0.587	175	217	41	22	2	1
Benjafield	2000	Australia	Caucasian	91/	149	7	0.314	40	70	43	68	8	11
Shoji	2000	Japan	Asian	183/	193	7	0.462	139	164	41	27	3	2
KARVONEN	2002	Finland	Caucasian	505/	519	9	0.820	244	262	220	215	41	42
Di	2002	China	Asian	95/	95	7	0.511	70	83	25	12	0	0
Liu	2002	China	Asian	103/	74	7	0.205	54	55	44	19	5	0
Jia	2002	China	Asian	116/	136	8	0.316	83	114	29	20	4	2
Tan	2003	China	Asian	112/	112	8	0.012	73	78	25	26	14	8
Li	2004	China	Asian	310/	151	8	0.902	226	126	81	24	3	1
Xu	2004	China	Asian	203/	190	8	0.854	165	141	37	45	1	4
Djuric´	2005	Serbia	Caucasian	172/	200	7	0.782	84	93	71	88	17	19
Moe	2006	Singapore	Asian	103/	104	7	0.787	79	82	20	21	4	1
Marcun-Varda	2006	Slovenia	Caucasian	104/	200	7	0.901	43	74	49	96	12	30
Dong	2006	China	Asian	97/	87	7	0.983	41	62	50	23	6	2
Ma	2006	China	Asian	192/	122	7	0.274	76	46	89	53	27	23
Wang	2006	China	Asian	277/	547	7	0.284	233	468	40	74	4	5
Zhang	2006	China	Asian	375/	414	7	< 0.001	212	273	106	93	57	48
Liang	2006	China	Asian	124/	100	8	0.625	108	85	11	14	5	1
Zhang	2006	China	Asian	190/	94	8	0.791	164	89	19	5	7	0
Zhao	2006	China	Asian	501/	489	7	0.692	404	387	93	97	4	5
Khawaja	2007	Pakistan	Mixed	143/	184	6	0.689	99	129	37	51	7	4
Wang	2007	China	Asian	100/	50	7	0.101	70	44	27	5	3	1
Colomba	2008	Italy	Caucasian	127/	67	7	0.030	45	19	70	41	12	7
Nejatizadeh	2008	India	Asian	453/	344	7	0.006	259	222	118	98	76	24
Periaswamy	2008	India	Asian	438/	444	8	0.656	291	323	126	110	21	11
Srivastava	2008	India	Asian	226/	200	8	0.556	139	154	82	44	5	2
Ghazali	2008	Malaysia	Asian	200/	198	8	0.920	144	151	54	44	2	3
Tang	2008	China	Asian	184/	196	6	0.983	91	95	80	83	13	18
Zhao	2008	China	Asian	174/	112	7	0.733	138	105	32	7	4	0
Tang	2008	China	Asian	271/	267	6	< 0.001	171	169	73	65	27	33
Wang	2009	China	Asian	230/	186	8	0.518	9	12	46	64	175	110
Zhang	2009	China	Asian	349/	214	8	0.267	260	179	79	32	10	3
Liu	2009	China	Asian	129/	117	7	0.311	76	85	46	31	7	1
Niu	2009	China	Asian	1305/	1154	8	0.008	1071	954	192	182	42	18
Kitsios	2010	Greece	Caucasian	228/	302	6	0.512	99	135	95	130	34	37
Wang	2010	China	Asian	154/	150	8	0.240	98	116	40	30	16	4
Zhou	2010	China	Asian	176/	131	6	0.351	137	98	38	32	1	1
Souza-Costa	2011	Brazil	Mixed	73/	285	8	0.086	45	172	25	105	3	8
Zhou	2011	China	Asian	346/	385	8	0.667	280	312	62	70	4	3
Chen	2011	China	Asian	160/	176	8	0.161	138	154	21	20	1	2
Zhao	2011	China	Asian	100/	97	8	0.648	96	82	3	14	1	1
Li	2011	China	Asian	510/	510	7	< 0.001	320	367	129	89	61	54
Ma	2012	China	Asian	300/	288	8	0.577	255	250	43	36	2	2
Zhang	2012	China	Asian	363/	370	6	0.580	265	278	85	84	13	8
Liang	2012	China	Asian	350/	150	7	0.965	290	127	57	22	3	1
Li	2012	China	Asian	227/	359	7	0.549	185	296	40	61	2	2
Goncharov	2013	Ukraine	Caucasian	145/	144	7	< 0.001	65	45	60	93	20	6
Yan	2013	China	Asian	308/	181	8	0.105	235	142	57	34	16	5
Yang	2013	China	Asian	134/	115	6	0.791	70	97	59	17	5	1
Ogretmen	2014	Turkey	Caucasian	21/	109	6	0.746	7	70	13	34	1	5
Shankarishan	2014	India	Caucasian	350	/350	8	0.261	194	296	133	50	23	4
Cui	2014	China	Asian	172	/90	8	0.786	133	85	36	5	3	0
Liu	2014	China	Asian	215	/108	8	0.283	149	89	48	17	18	2
Hui	2015	China	Asian	100	/100	6	0.677	81	92	16	8	3	0
Xiong	2015	China	Asian	226	/186	8	0.752	130	133	83	48	13	5
ALrefai	2016	Egypt	Caucasian	70	/30	7	0.773	49	27	16	3	5	0
Gamil	2017	Sudan	Caucasian	147	/82	6	0.829	100	60	42	20	5	2
Zhang	2017	China	Asian	456	/453	8	0.001	365	362	84	78	7	13
Nassereddine	2018	Morocco	Caucasian	145	/184	6	0.509	5	116	54	62	86	6

### Association between eNOS rs1799983 polymorphism and hypertension

**Fig. 2:**
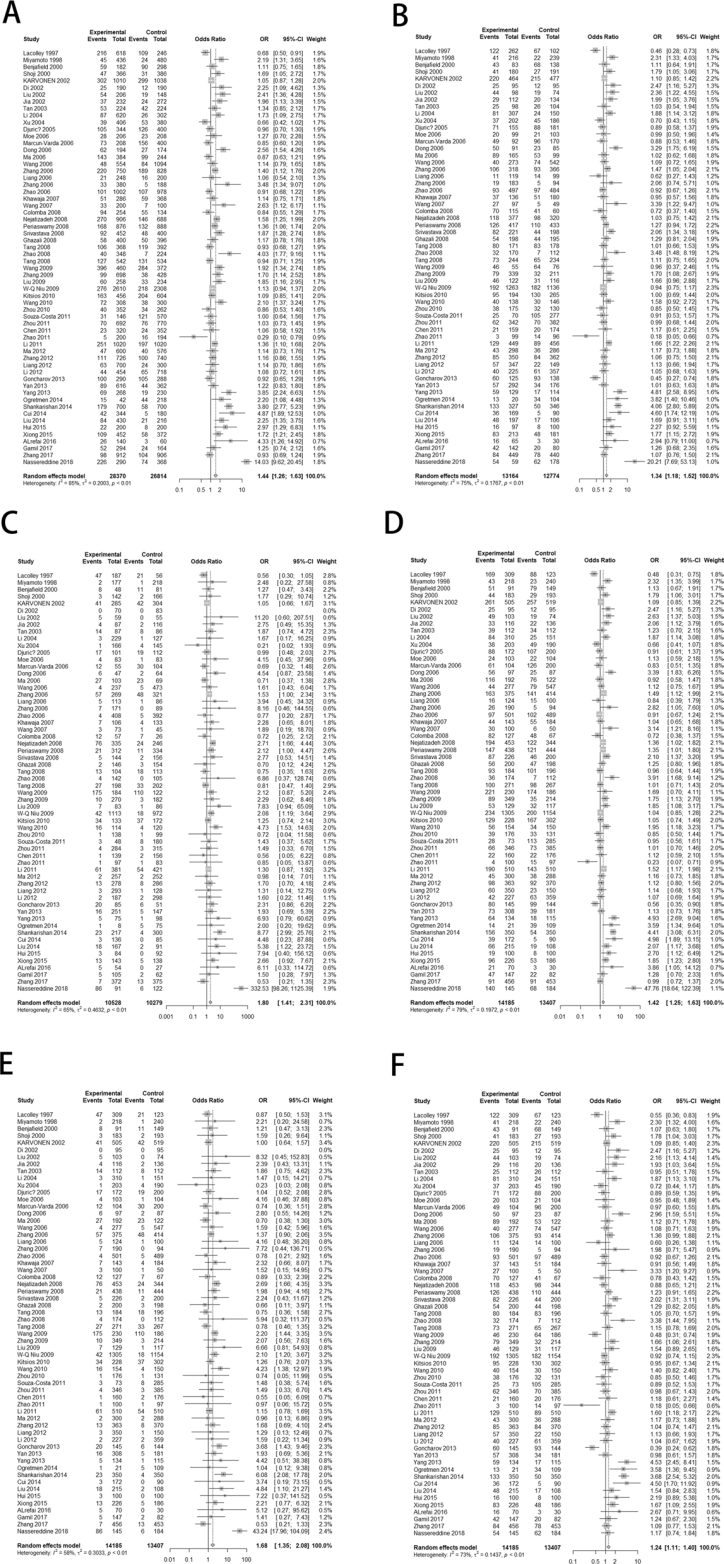
Forest plot for the result of association between *eNOS* rs1799983 polymorphism and hypertension based on a random-effects model. **A** Allelic model: T vs G; **B** codominant model: GT vs GG; **C** codominant model: TT vs GG; **D** dominant model: GT + TT vs GG; **E** recessive model: TT vs GG + GT; **F** overdominant model: GT vs GG + TT

There were significant heterogeneities between *eNOS* rs1799983 polymorphism and hypertension in the five different genetic models, and thus random-effects model was used for all analyses. We found significant association between *eNOS* rs1799983 polymorphism and the risk of hypertension under any genetic model (T vs G: OR 1.44, 95% CI 1.26–1.63; GT vs GG: OR 1.34, 95% CI 1.18–1.52; TT vs GG: OR 1.80, 95% CI 1.41–2.31; GT + TT vs GG: OR 1.42, 95% CI 1.25–1.63; TT vs GG + GT: OR 1.68, 95% CI 1.35–2.08; GT vs GG + TT: OR 1.24, 95% CI 1.11–1.40) (Fig. 2).

### Subgroup analysis

We performed subgroup analysis by region and ethnicity because gene polymorphism may be associated with variations in region and ethnicity. For region, there is only difference for the association between *eNOS* rs1799983 polymorphism and hypertension under overdominant model, when GT was compared with GG + TT, the association with risk of hypertension was identified in China (OR 1.29; 95% CI 1.12–1.49), and the association between *eNOS* rs1799983 polymorphism with risk of hypertension was found in any region under other genetic models. With regard to ethnicity, we found the association between *eNOS* rs1799983 polymorphism with risk of hypertension was significant in Asian population under all genetic models (T vs G: OR 1.42, 95% CI 1.27–1.58; GT vs GG: OR 1.37, 95% CI 1.21–1.54; TT vs GG: OR 1.64, 95% CI 1.35–2.00; GT + TT vs GG: OR 1.43, 95% CI 1.27–1.61; TT vs GG + GT: OR 1.56, 95% CI 1.29–1.88; GT vs GG + TT: OR 1.31, 95% CI 1.15–1.48); however, with respect to contrast of TT versus GG and TT versus GG + GT, the genotype TT was associated with the increased risk of hypertension not only in Asian population but also in other population (OR 2.07, 95% CI 1.05–4.08 and OR 1.87, 95% CI 1.07–3.25, respectively) (Table 2).

**Table 2:** Overall and subgroup analysis of association between *eNOS* rs1799983 polymorphism and hypertension under different models

Categories	T versus G			GT versus GG			TT versus GG			GT + TT versus GG			TT versus GG + GT			GT versus GG + TT		
	OR	(95% CI)	*I*^2^ (%)	OR	(95%CI)	*I*^2^ (%)	OR	(95%CI)	*I*^2^ (%)	OR	(95%CI)	*I*^2^ (%)	OR	(95%CI)	*I*^2^ (%)	OR	(95%CI)	*I*^2^ (%)
Overall	**1.44**	(1.26,1.63)	85	**1.34**	(1.18,1.52)	75	**1.80**	(1.41,2.31)	65	**1.42**	(1.25,1.63)	79	**1.68**	(1.35,2.08)	58	**1.24**	(1.11,1.40)	73
Region																		
China	**1.40**	(1.23,1.59)	72	**1.35**	(1.18,1.55)	65	**1.54**	(1.24,1.93)	24	**1.42**	(1.23,1.63)	69	**1.47**	(1.19,1.81)	24	**1.29**	(1.12,1.49)	67
Other	**1.47**	(1.12,1.91)	92	**1.31**	(1.01,1.71)	85	**2.05**	(1.24,3.40)	82	**1.44**	(1.09,1.89)	87	**1.89**	(1.24,2.88)	77	1.16	(0.94,1.44)	79
Ethnicity																		
Asian	**1.42**	(1.27,1.58)	69	**1.37**	(1.21,1.54)	63	**1.64**	(1.35,2.00)	23	**1.43**	(1.27,1.61)	66	**1.56**	(1.29,1.88)	23	**1.31**	(1.15,1.48)	66
Other	1.44	(0.98,2.12)	94	1.28	(0.87,1.87)	88	**2.07**	(1.05,4.08)	88	1.42	(0.94,2.15)	91	**1.87**	(1.07,3.25)	83	1.07	(0.80,1.43)	83

The significance of bold: *P*<0.05

### Sensitivity analysis and publication bias

To examine the influence of individual study on the overall results, sensitivity analysis was performed by excluding a single study at each time in our meta-analysis. The results of sensitivity analysis showed that the corresponding pooled *ORs* and 95% CIs under any model of inheritance were not substantially altered after excluding any single study, suggesting that results of our meta-analysis were relatively stable and credible (Additional file 2: Figure S1).

Publication bias was evaluated by funnel plots and quantified by Egger’s tests. The funnel plots for recessive model (TT vs GG + GT) seemed symmetrical, and the results of Egger’s tests showed that there was no publication bias (*P* = 0.102); however, the funnel plots were asymmetrical in other genetic models for the association between *eNOS* rs1799983 polymorphism with hypertension, and the results of Egger’s tests showed that there were publication bias (T vs G: *P* = 0.026; GT vs GG: *P* = 0.023; TT vs GG: *P* = 0.032; GT + TT vs GG: *P* = 0.011; GT vs GG + TT: *P* = 0.038) (Additional file 3: Figure S2).

### Trial sequential analysis (TSA)

**Fig. 3:**
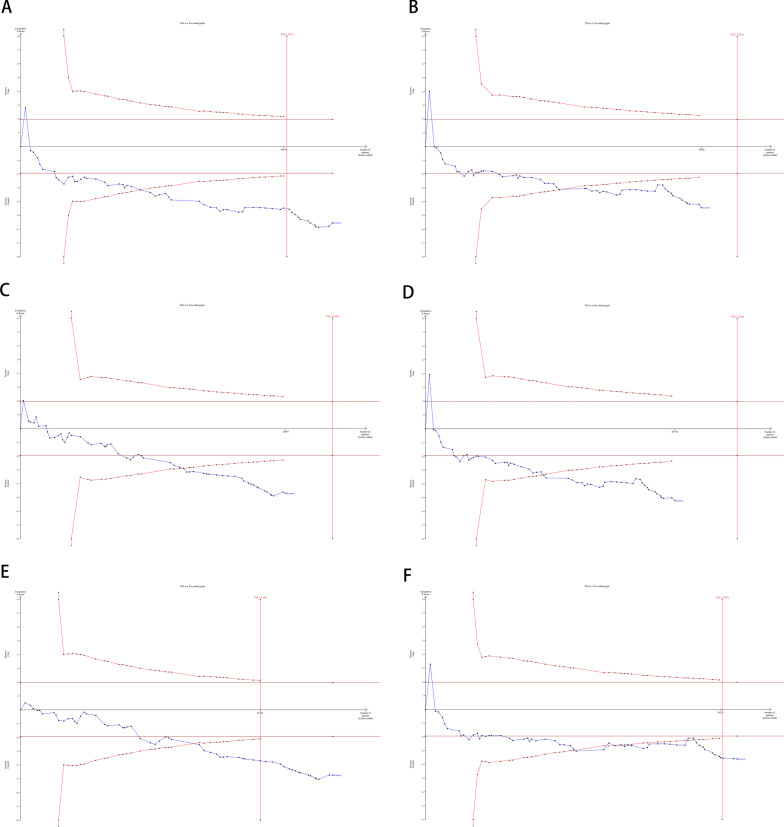
Trial sequential analysis of association between *eNOS* rs1799983 polymorphism and hypertension. **A** Allelic model: T vs G; **B** codominant model: GT vs GG; **C** codominant model: TT vs GG; **D** dominant model: GT + TT vs GG; **E** recessive model: TT vs GG + GT; **F** overdominant model: GT vs GG + TT

For the association between *eNOS* rs1799983 polymorphism with hypertension under codominant model (GT vs GG), codominant model (TT vs GG), and dominant model (GT + TT vs GG), the Z-curve crossed trial sequential monitoring boundary, although the sample size did not reach the RIS (Fig. 3B–D). However, for the association between *eNOS* rs1799983 polymorphism with hypertension under allelic model (T vs G), recessive model (TT vs GG + GT), and overdominant model (GT vs GG + TT), the Z-curve crossed trial sequential monitoring boundary, and the sample sizes were also more than the RIS (Fig. 3A, E, F). Therefore, concrete evidence indicates that further studies are not necessary for the association between *eNOS* rs1799983 polymorphism with hypertension.

## Discussion

In the meta-analysis, we collected related articles comprehensively to investigate the association between *eNOS* rs1799983 polymorphism and hypertension. Our results suggest that there is an association between *eNOS* rs1799983 polymorphism and risk of hypertension under any genetic model (T vs G, GT vs GG, TT vs GG, GT + TT vs GG, TT vs GG + GT, and GT vs GG + TT), especially among Asian population. Moreover, with respect to contrast of TT versus GG and TT versus GG + GT, the TT genotype is associated with the increased risk of hypertension not only in Asian population but also in other population.

Nine meta-analyses on association between *eNOS* rs1799983 polymorphism and hypertension have been published, four of them (Chen et al., Wang et al., Li et al., and Liu et al.) studied the Chinese populations [20–23]. Chen et al. and Wang et al. studied the two models (T vs G and GT + TT vs GG) of our models in this meta-analysis, and their results are consistent with our results, we all found that T allele and GT + TT genotype are associated with an increased risk of hypertension. In addition, Li et al. studied the association between T allele of *eNOS* rs1799983 polymorphism and hypertension, and Liu et al. studied the association between GT + TT genotype of *eNOS* rs1799983 polymorphism and hypertension, and their results are also consistent with our results.

Pereira et al. [24] studied the association between GT + TT genotype of *eNOS* rs1799983 polymorphism and hypertension, and consistent with the discoveries of Pereira et al., we also identified the heterogeneity and publication bias in the meta-analysis, they may exist owing to the gene–environment interactions. Niu et al. [25] only studied the association between T allele of *eNOS* rs1799983 polymorphism and hypertension, we all found the T allele of *eNOS* rs1799983 polymorphism was a risk factor of hypertension, especially among Asian population. Moreover, of the nine meta-analyses, the results of Takeuchi [26] and Zintzaras [27] were negative, they found that there was no association between *eNOS* rs1799983 polymorphism and hypertension, the reason they had this negative results may be a small size, or interaction of polymorphisms within haplotypes, which is a major determinant of disease susceptibility, not the individual polymorphism [28].

For the meta-analysis of Xie et al. [13], the last meta-analysis published in 2017, their results showed there is no association between TT genotype and hypertension when TT genotype was compared with GG + GT genotype, but TT genotype was associated with the increased risk of hypertension in our meta-analysis. In addition, our result of TSA also demonstrated that the Z-curve crossed trial sequential monitoring boundary, and the sample sizes were also more than the RIS. Therefore, it is adequate to draw a conclusion that TT genotype is associated with the increased risk of hypertension.

The meta-analysis may report false positive results for the risk of type I errors, and these results are usually caused by publication bias, heterogeneity between studies, or poor study quality. However, a limited number of trials may not provide enough information, resulting in incorrect estimates [29]. Thus, we conducted TSA to reduce the risk of type I errors and evaluated whether further studies are necessary by calculating the required information size. In our meta-analysis, either the sample size was greater than the required information size or the Z-curve crossed trial sequential monitoring boundary, indicating that the results of our meta-analysis are reliable and sufficient to draw conclusions on the association between *eNOS* rs1799983 polymorphism and hypertension.

The vasodilator effect of NO that is produced by eNOS is very important for maintenance of vascular function [30], and the G894T polymorphism (Glu298Asp or rs1799983) at exon 7 of the eNOS gene is associated with reduced eNOS expression, activity and subsequently reduced NO production, could be a potential candidate marker for hypertension development [31, 32]. Moreover, clinical studies have showed that vascular responsiveness is altered in subjects with this variant owing to an increased vasoconstrictive response to phenylephrine for the subjects with Asp298 [33], and several clinical and experimental studies also indicate that alteration of NO metabolism plays a key role in the occurrence and conventional therapy of hypertension [34–36].Therefore, it is necessary to identify the association between *eNOS* rs1799983 polymorphism and hypertension.

Our study has some limitations. First, there is heterogeneity in our article, and the main sources of heterogeneity remain unclear. Second, publication bias was found in the association between *eNOS* rs1799983 polymorphism and hypertension under any genetic model except the recessive model, because negative articles are unpublished. Third, our research cannot prove the existence of causality, but only an association because of the design of case–control.

Despite the above limitations, our research also has some advantages. First of all, we have collected the latest articles extensively, which provides more statistical power to draw effective conclusions on this issue. Secondly, the results of sensitivity analysis show that our conclusion is stable and reliable. Third, to our knowledge, this is the first TSA to evaluate the association between *eNOS* rs1799983 polymorphism and hypertension, which further offers reliable evidence to reach the conclusion.

## Conclusion

In conclusion, *eNOS* rs1799983 polymorphism is associated with increased risk of hypertension under any genetic model. Moreover, investigations of gene–gene and gene–environment interactions are needed to give more insight into the association between *eNOS* rs1799983 polymorphism and hypertension.

## Supplementary Information

**Additional file 1. Table S1** Search strategies of databases.

**Additional file 2. Figure S1** Sensitivity analysis of association between eNOS rs1799983 polymorphism and hypertension. (A) allelic model: T vs G; (B) codominant model: GT vs GG; (C) codominant model: TT vs GG; (D) dominant model: GT + TT vs GG; (E) recessive model: TT vs GG + GT; (F) overdominant model: GT vs GG + TT.

**Additional file 3. Figure S2** Funnel plot for the result of association between eNOS rs1799983 polymorphism and hypertension. (A) allelic model: T vs G; (B) codominant model: GT vs GG; (C) codominant model: TT vs GG; (D) dominant model: GT+TT vs GG; (E) recessive model: TT vs GG + GT; (F) overdominant model: GT vs GG + TT

## Data Availability

All data generated or analysed during this study are included in this published article.

## Abbreviations

Asp: Aspartic acid
BP: Blood pressure
CI: Confidence intervals
EH: Essential hypertension
*eNOS*: Encoding endothelial nitric oxide synthase
Glu: Glutamic acid
HWE: Hardy–Weinberg Equilibrium
NOS: Newcastle–Ottawa scale
NO: Nitric oxide
OR: Odds ratios
TSA: Trial sequential analysis
RIS: Required information size
