# Supplementary material for: Association between eNOS rs1799983 polymorphism and hypertension: a meta-analysis involving 14,185 cases and 13,407 controls
Source: BMC Cardiovasc Disord. 2021 Aug 9;21:385. doi: 10.1186/s12872-021-02192-2 (PMC8351409; doi:10.1186/s12872-021-02192-2)
Supplement: Supplementary file 1 — Additional file 1. Table S1 Search strategies of databases. [file 12872_2021_2192_MOESM1_ESM.docx]

Table S1. Search strategies of databases.

| Datebase | Search strategies |
| --- | --- |
| PubMed, Embase, Web of Science, Medline, Scopus | (“endothelial nitric oxide synthase” OR “nitric oxide synthase type III” OR “*eNOS*” OR “*NOS3*”) AND (“polymorphism” OR ‘‘variant” OR “mutation”) AND (“hypertension” OR “high blood pressure”) |
| WanFang datebase, Vip datebase,  CNKI database | The equivalent Chinese terms were used in the Chinese databases |
