# Supplementary figures and images for: Association between eNOS rs1799983 polymorphism and hypertension: a meta-analysis involving 14,185 cases and 13,407 controls
Source: BMC Cardiovasc Disord. 2021 Aug 9;21:385. doi: 10.1186/s12872-021-02192-2 (PMC8351409; doi:10.1186/s12872-021-02192-2)

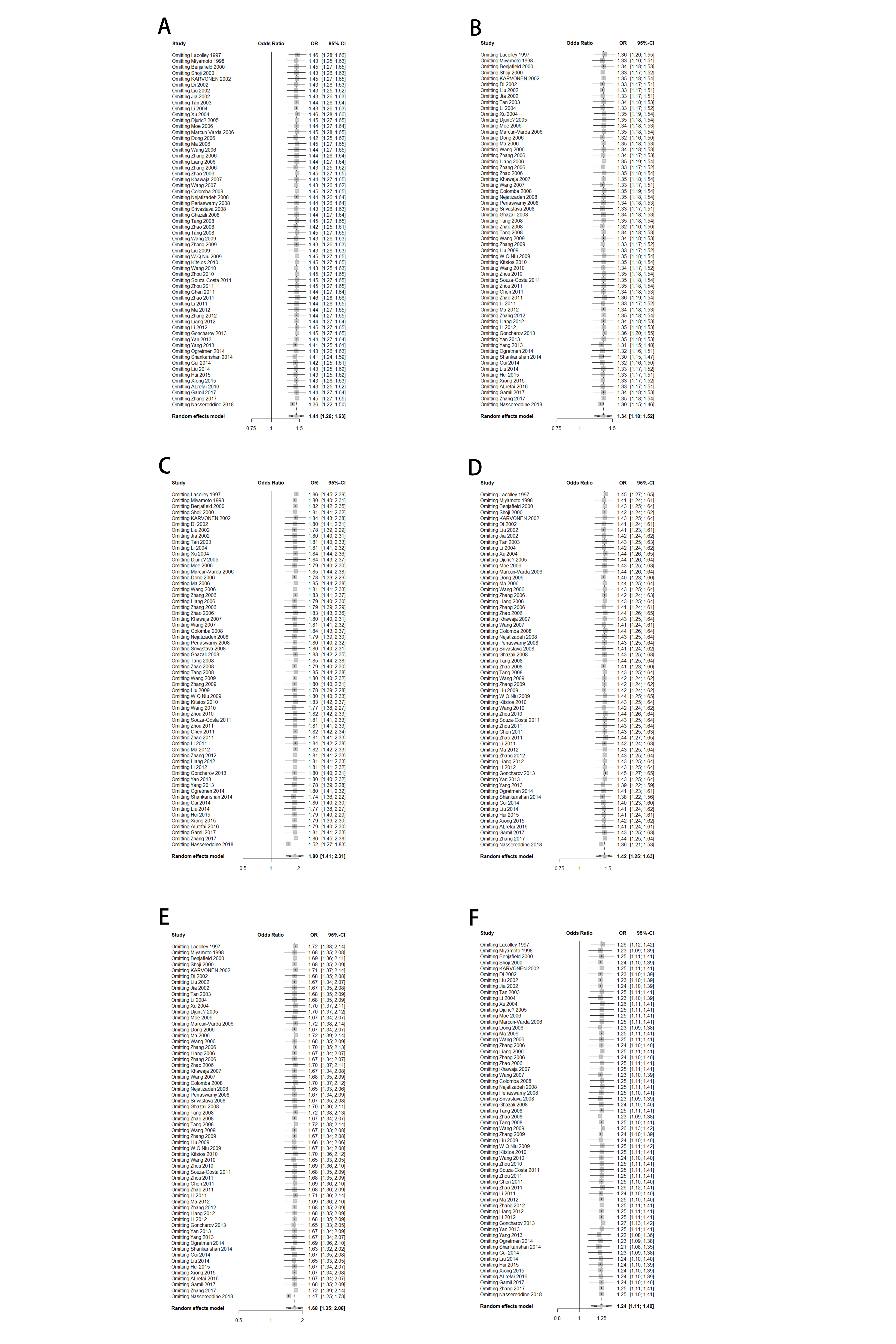

Supplement: Supplementary file 2 — Additional file 2. Figure S1 Sensitivity analysis of association between eNOS rs1799983 polymorphism and hypertension. (A) allelic model: T vs G; (B) codominant model: GT vs GG; (C) codominant model: TT vs GG; (D) dominant model: GT + TT vs GG; (E) recessive model: TT vs GG + GT; (F) overdominant model: GT vs GG + TT. [file 12872_2021_2192_MOESM2_ESM.tif]

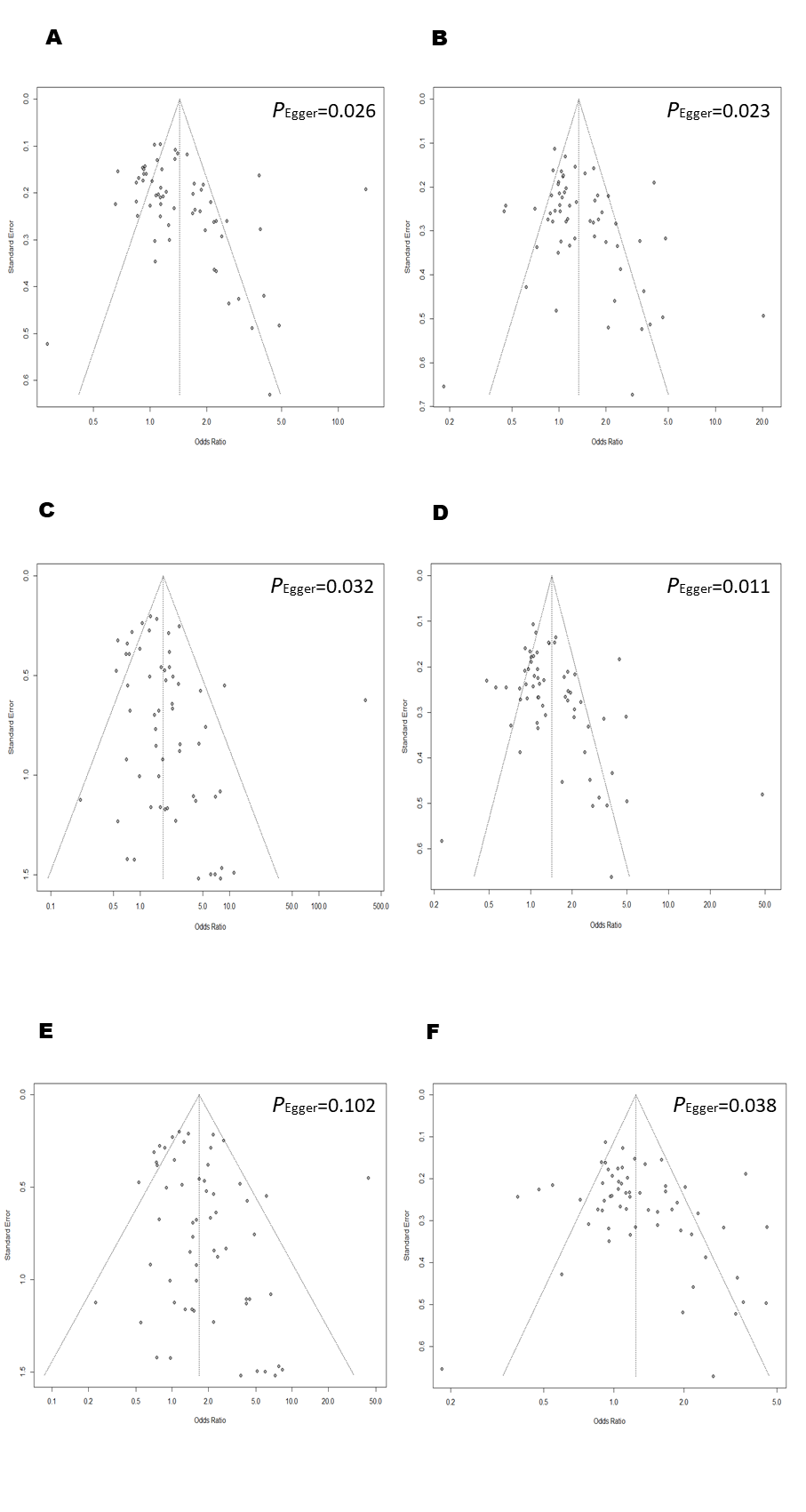

Supplement: Supplementary file 3 — Additional file 3. Figure S2 Funnel plot for the result of association between eNOS rs1799983 polymorphism and hypertension. (A) allelic model: T vs G; (B) codominant model: GT vs GG; (C) codominant model: TT vs GG; (D) dominant model: GT+TT vs GG; (E) recessive model: TT vs GG + GT; (F) overdominant model: GT vs GG + TT [file 12872_2021_2192_MOESM3_ESM.tif]
